# Prognostic value, localization and correlation of PD-1/PD-L1, CD8 and FOXP3 with the desmoplastic stroma in pancreatic ductal adenocarcinoma

**DOI:** 10.18632/oncotarget.10038

**Published:** 2016-06-14

**Authors:** Angela Diana, Lai Mun Wang, Zenobia D'Costa, Paul Allen, Abul Azad, Michael A. Silva, Zahir Soonawalla, Stanley Liu, W. Gillies McKenna, Ruth J. Muschel, Emmanouil Fokas

**Affiliations:** ^1^ Department of Oncology, CRUK/MRC Oxford Institute for Radiation Oncology, University of Oxford, Oxford, UK; ^2^ Department of Pathology, Oxford University Hospital NHS Foundation Trust, Oxford, UK; ^3^ Department of Surgery, Oxford University Hospital NHS Foundation Trust, Oxford, UK; ^4^ Department of Radiation Oncology, Sunnybrook Research Institute, Sunnybrook Health Sciences Centre, University of Toronto, Toronto, Canada

**Keywords:** PD-1/PD-L1, CD8, immune, prognosis, pancreatic cancer, Immunology and Microbiology Section, Immune response, Immunity

## Abstract

We examined the prognostic value of programmed cell death-1 (PD-1) and its ligand (PD-L1) together with CD8+ tumor-infiltrating lymphocytes (TILs) and FOXP3+ Tregs in resectable pancreatic ductal adenocarcinoma (PDAC) samples treated with adjuvant chemotherapy. Whole-mount FFPE tissue sections from 145 pancreatectomies were immunohistochemically stained for PD-1, PD-L1, CD8 and FOXP3. Their expression was correlated with clinicopathological characteristics, and overall survival (OS), progression-free survival (PFS), local progression-free survival (LPFS) and distant metastases free-survival (DMFS), in the context of stroma density (haematoxylin-eosin) and activity (alpha-smooth muscle actin) and in regard to intratumoral lymphoid aggregates. The median OS was 21 months after a mean follow-up of 20 months (range, 2-69 months). In multivariate analysis, high PD-1+ TILs expression was associated with better OS (*p* = 0.049), LPFS (*p* = 0.017) and DMFS (*p* = 0.021). Similar findings were observed for CD8+ TILs, whereas FOXP3 and PD-L1 lacked prognostic significance. Although TIL distribution was heterogeneous, tumors of high stroma density had higher infiltration of CD8+ TILs than loose density stroma and vice versa (*p* < 0.001), whereas no correlation was found with stromal activity. Sixty (41.4%) tumors contained lymphoid aggregates and the presence of PD-1+ TILs was associated with better OS (*p* = 0.030), LPFS (*p* = 0.025) and DMFS (*p* = 0.033), whereas CD8+ TILs only correlated with superior LPFS (*p* = 0.039). PD-1+ and CD8+ TILs constitute independent prognostic markers in patients with PDAC treated with adjuvant chemotherapy. Our study provides important insight on the role of PD-1/PD-L1 in the context of desmoplastic stroma and could help guide future immunotherapies in PDAC.

## INTRODUCTION

Pancreatic ductal adenocarcinoma (PDAC) is a highly lethal malignancy with a 5-year survival of approximately 5% [1, 2]. Although surgery constitutes the only potentially curative treatment for PDAC, most patients who have undergone surgery still develop recurrences and additional treatments, such as chemotherapy and radiotherapy only offer modest benefit [1, 2]. PDAC is characterised by an abundant desmoplastic stroma composed of activated stellate cells, extracellular matrix, immunosuppressive cell populations, such as regulatory T cells (Tregs), and limited protective immunosurveillance due to the low number of cytotoxic CD8+ tumor infiltrating lymphocytes (TILs) observed in the majority of the patients [3, 4].

Program death receptor-1 (PD-1) and its ligand, program death ligand 1 (PD-L1), mediate immune tolerance PD-L1 is typically expressed on cancer cells, parenchymal and myeloid cells, whereas PD-1 is expressed on activated T cells [5, 6]. Binding of PD-1 to PD-L1 leads to T cell anergy or exhaustion that impairs anti-tumor immune responses, resulting in tumor growth and progression. PD-L1 is upregulated in malignancies due to either activated oncogenic signalling, such as the PI3K/Akt pathway (innate resistance) or in response to interferon gamma secretion by T cells (adaptive resistance) [5, 6].

Immune checkpoint inhibitors targeting the PD-1/PD-L1 pathway and the cytotoxic T-lymphocyte antigen-4 (CTLA-4) have shown impressive rates of durable clinical responses in patients with melanoma, renal cell carcinoma, non-small lung cancer [6] but administration of these agents as monotherapy in PDAC failed to demonstrate anti-tumor activity [7, 8] despite initially promising preclinical findings [9]. There are plausible explanations for the lack of efficacy of these agents in PDAC including the low level of tumor infiltration by effector immune cells [4, 10, 11] and the presence of fibroblast activation protein (FAP)-positive mesenchymal stromal cells that mediate immunosuppression via the CXCR4/CXCL-12 chemokine pathway [12].

The level of CD8+ TILs and FOXP3+ regulator Tregs has been correlated with the clinical outcome in several malignancies [13]. Although recent studies have investigated the prognostic impact of CD8+ TILs and FOXP3 Tregs in PDAC [11], the prognostic role and correlation with PD-1+ TILs and PD-L1+ cell expression in this disease remains largely unknown. Also, the role of these immune markers in the context of the desmoplastic stroma has not been investigated. We aimed to evaluate the prognostic significance of the CD8, FOXP3, PD-1 and PD-L1 expression alone, and also the correlation with the desmoplastic stroma density and activity based on haematoxylin-eosin and αSMA, respectively, in a relatively large number of patients (n=145) treated with surgery followed by adjuvant chemotherapy. Because 60 (41.4%) patients from the entire cohort presented intratumoral lymphoid aggregates i.e. ectopic lymph-node like structures, we also evaluated the expression and prognostic role of CD8, FOXP3, PD-1 and PD-L1 expression in the lymphoid aggregates separately. Importantly, in contrast to the majority of previous studies that used tissue microarrays or small sections, we conducted our analyses on large sections from the entire pancreatectomy specimen allowing a more complete assessment of tissue heterogeneity and localization of the immune markers.

## RESULTS

### Immune markers staining characteristics

**Figure 1:**
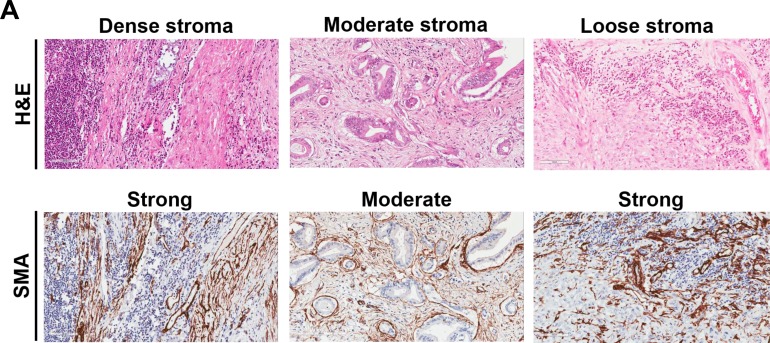

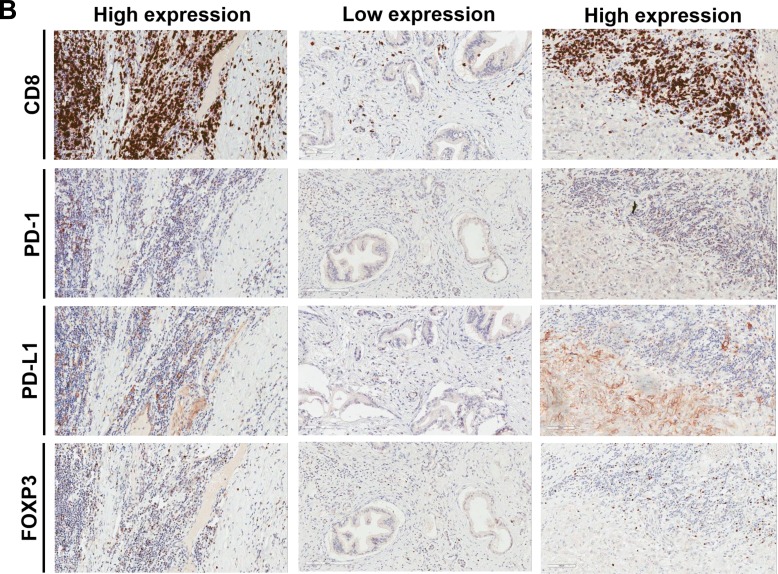
Representative examples of immune cells and markers in pancreatic cancer adenocarcinoma based on tumor stroma density **A.** H&E staining shows examples of tumors with high-density, moderate density, and low density stroma, as indicated. The corresponding αSMA images are shown as well. **B.** CD8+ TILs, PD-1+ TILs, PD-L1 tumor (and myeloid cells) and FOXP3+ Tregs staining from the same sections shown in Figure 1A, as indicated. Magnification, x200.

The results of CD8, FOXP3, PD-1 and PD-L1 immunohistochemistry including the three individual tumor compartment scores (intraepithelial, stroma and periphery) and the total score from all compartments are shown in Table 1. Regarding the correlation of the immune markers with the clinicopathologic characteristics (Table 2), tumors high PD-1+ TILs expression were significantly associated with more advanced T-stage (T3-4 vs T1-2; p=0.002). High PD-L1 expression correlated with lower T-stage (p=0.007). We failed to identify any further significant relationship between immune markers expression and clinicopathologic parameters (Table 2). Representative examples of low and high CD8+ and PD-1+ TILs as well as FOXP3+ Tregs and PD-L1+ tumor cells are shown in three different cases with either high, moderate or loose stroma density in Figure 1 and Supplementary Figure 1. The clinicopathologic characteristics for the entire cohort are depicted in Supplementary Table 1.

**Table 1:** Results of CD8, FOXP3, PD-1 and PD-L1 immunohistochemistry

Immune marker	CD8 *n* (%)	FOXP3 *n* (%)	PD-1 *n* (%)	PD-L1 *n* (%)
Dichotomized total score^*^	<6 *vs* ≥6	<4 *vs* ≥4	<3 *vs* ≥3	<3 *vs* ≥3
Low total score	85 (58.6)	38 (26.2)	79 (54.5)	104 (71.7)
High total score	60 (41.4)	107 (73.8)	66 (45.5)	41 (28.3)
Dichotomized intraepithelial compartment score^*^	≤2 *vs* >2	1 *vs* 2	1 *vs* 2	1 *vs* ≥2
Low total score	72 (49.7)	39 (26.9)	130 (89.7)	118 (81.4)
High total score	73 (50.3)	106 (73.1)	15 (10.3)	27 (18.6)
Dichotomized tumor stroma compartment score^*^	≤2 *vs* >2	≤2 *vs* >2	1 *vs* ≥2	1 *vs* ≥2
Low total score	115 (79.3)	38 (26.2)	93 (64.1)	116 (80)
High total score	30(20.7)	107 (73.8)	52 (35.9)	29 (20)
Dichotomized tumor periphery compartment score^*^	1 *vs* ≥2	1 *vs* 2	1 *vs* 2	1 *vs* ≥2
Low total score	60 (59.4)	37 (25.5)	105 (72.4)	118 (81.4)
High total score	41(40.6)	108 (74.5)	40 (27.6)	27 (18.6)

* Dichotomized labelling (low vs high score) based on the median value of immune marker expression. Total score accounted for all three compartment scores (intraepithelial, stroma, periphery).

**Table 2:** Clinicopathological characteristics for the entire cohort (n=145)

	Low CD8 n.(%)	High CD8 n (%)	p-value	low FOXP3 n (%)	High FOXP3 n (%)	p-value	low PD-1 n (%)	High PD-1 n (%)	p-value	low PD-L1 n (%)	High PD-L1 n (%)	p-value
**Age**												
<median (65 years)	41 (48.2%)	22 (36.7%)	0.166	18 (47.4%)	45 (42.1%)	0.570	39 (49.4%)	24 (36.4%)	0.116	47 (45.2%)	16 (39%)	0.500
≥median	44 (51.8%)	38 (63.3%)		20 (56.2%)	62 (57.9%)		40 (50.6%)	42 (63.6%)		57 (54.8%)	25 (61%)	
**Gender**												
Female	35 (41.2%)	33 (55.0%)	0.100	13 (34.2%)	55 (51.4%)	0.068	36 (45.6%)	32 (48.5%)	0.726	44 (43.2%)	24 (58.5%)	0.114
Male	50 (58.8%)	27 (45.0%)		25 (65.8%)	52 (48.6%)		43 (54.4%)	34 (51.5%)		60 (57.7%)	17 (41.5%)	
**Tumor site**												
Head	66 (77.6%)	54 (90%)	0.086	32 (84.2%)	88 (82.2%)	0.783	69 (87.3%)	51 (77.3%)	0.110	85 (81.7%)	35 (85.4%)	0.602
Other	19(22.4%)	6 (10%)		6 (15.8%)	19 (17.8%)		10 (12.7%)	15 (22.7%)		19 (18.3%)	6 (14.6%)	
**pT.staging**												
pT1-2	54 (63.5%)	34 (56.7%)	0.405	26 (68.4%)	62 (57.9%)	0.256	57 (72.2%)	31 (47.0%)	**0.002**	56 (53.8%)	32 (78%)	**0.007**
pT3-4	31 (36.5%)	26 (43.3%)		12 (31.6%)	45 (42.1%)		22 (27.8%)	35 (53%)		48 (46.2%)	9 (22%)	
**pN. staging**												
pNO	17 (20%)	18 (30%)	0.166	11 (28.9%)	24 (22.4%)	0.420	16 (20.3%)	19 (28.8%)	0.232	23 (22.1%)	12 (29.3%)	0.365
pN+	68 (80%)	42 (70%)		27 (71.1%)	83 (77.6%)		63 (79.7%)	47 (71.2%)		81 (77.9%)	29 (70.7%)	
**Grading**												
G1	4 (4.7%)	4 (6.7%)	0.203	2 (5.3%)	6(5.6%)	0.300	4 (5.1%)	4 (6.1%)	0.835	6(5.8%)	2 (4.9%)	0.518
G2	51 (60%)	43 (71.7%)		21 (55.3%)	73 (68.2%)		50 (63.3%)	44 (66.7%)		70 (67.3%)	24 (58.5%)	
G3	30 (35.3%)	13 (21.7%)		15 (39.5%)	28 (26.2%)		25 (31.6%)	18 (27.3%)		28 (26.9%)	15 (36.6%)	
**Resection margins**												
RO	27 (31.8%)	27 (45%)	0.104	17 (44.7%)	37 (34.6%)	0.266	26 (32.9%)	28 (42.4%)	0.238	36 (34.6%)	18 (43.9%)	0.298
R1	58 (68.2%)	33 (55%)		21 (55.3%)	70 (65.4%)		53 (67.1%)	38 (57.6%)		68 (65.4%)	23 (56.1%)	
**Type of surgery**												
Whipples	49 (57.6%)	43 (71.7%)	0.120	26 (68.4%)	66 (61.7%)	0.732	52 (65.8%)	40 (60.6%)	0.590	65 (62.5%)	27 (65.9%)	0.753
Pylorus preserving	24 (28.2%)	14 (23.3%)		9 (23.7%)	29 (27.1%)		21 (26.6%)	17 (25.7%)		27 (26%)	11 (26.8%)	
Total pancreatectomy	12 (14.1%)	3(5%)		3(7.9%)	12 (11.2%)		6 (7.6%)	9 (13.6)		12 (11.5%)	3 (7. 3%)	
**PNI**												
no	68 (80%)	46 (76.7%)	0.630	28 (73.7%)	86 (80.4%)	0.388	58 (73.4%)	56 (84.8%)	0.095	81 (77.9%)	33 (80.5%)	0.731
yes	17 (20%)	14 (23.3%)		10 (26.3%)	21 (19.6%)		21 (26.6%)	10 (15.2%)		23 (22.1%)	8 (19.5%)	
**VI**												
no	29 (34.1%)	23 (38.3%)	0.602	14 (36.8%)	38 (35.5%)	0.883	29 (36.7%)	23 (34.8%)	0.816	37 (35.6%)	15 (36.6%)	0.909
yes	56 (65.9%)	37 (61.7%)		24 (63.2%)	69 (64.5%)		50 (63.3%)	43 (65.2%)		67 (64.4%)	26 (63.4%)	
**Ll**												
no	27 (31.8%)	26 (43.3%)	0.154	11 (28.9%)	42 (39.3%)	0.257	31 (39.2%)	22 (33.3%)	0.462	35 (33.7%)	18 (43.9%)	0.248
yes	58 (68.2%)	34 (56.7%)		27 (71.1%)	65 (60.7%)		48 (60.8%)	44 (66.7%)		69 (66.3%)	23 (56.1%)	
Chemotherapy												
No	13 (15.3%)	6 (10%)	0.343	5 (13.2%)	14 (13.1%)	0.541	11 (13.9%)	8 (12.1%)	0.506	12 (11.5%)	7(17.1%)	0.329
1-2 cycles	21 (24.7%)	11 (18.3%)		6(15.8%)	26 (24.3%)		20 (25.3%)	12 (18.2%)		26 (25%)	6(14.6%)	
≥ cycles	51 (60%)	43 (71.7)		27 (71.1%)	67 (62.6%)		48 (60.8%)	46 (69 7%)		66 (63.5%)	28 (68.3%)	

Abbreviations: HR, hazard ratio; Cl, confidence interval; VI, vascular invasion; Ll, lymphatic invasion; PNI, perineura1Jneural invasion;

### Immune markers and treatment response

**Figure 2:**
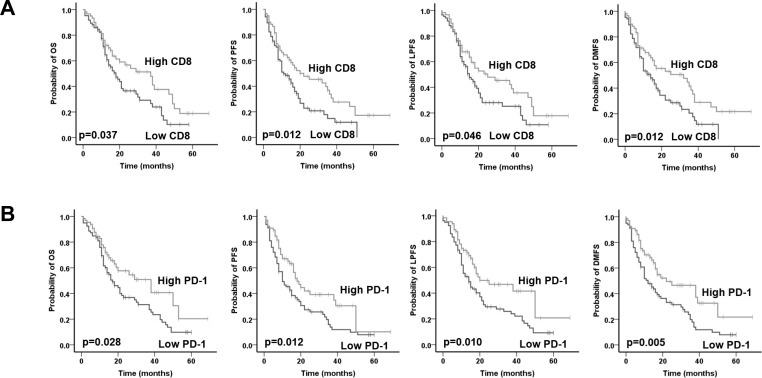
Prognostic impact of **A.** total CD8+ TILs and **B.** total PD-1+ TILs on overall survival (OS), progression-free survival (PFS), local progression-free survival (LPFS) and distant metastases free survival (DMFS) in in patients with pancreatic cancer adenocarcinoma, as indicated. Analysis was based on the dichotomized total CD8 and PD-1 score in resected patient samples (cut-off according to median value of total score).

The median OS for the entire cohort was 21 months and the 3-year OS was 35.7 % after a mean follow-up of 20 months (range, 2-69 months). In total, 56 (38.6%) patients developed distant recurrence, 15 (10.35%) developed local recurrence, 15 (10.35%) had both local and distant recurrence, whereas 59 (40.7%) had no recurrence by the time of analysis. Patients with high total CD8+ TILs expression had a significantly superior OS (low vs high CD8: mean 23.7 vs 33.8 months; p=0.046), PFS (low vs high CD8: mean 17.3 vs 28.8 months; p=0.012), LPFS (low vs high CD8: mean 21.8 vs 31.4 months; p=0.046) and DMFS (low vs high CD8: mean 18.8 vs 31.0 months; p=0.012) in univariate analysis (Figure 2A and Table 3). PD-1 was only expressed in TILs and patients with a high PD-1+ TILs expression had a significantly superior OS (low vs high PD-1: mean 24.1 vs 35.0 months; p=0.028), PFS (low vs high PD-1: mean 18.2 vs 27.6 months; p=0.012), LPFS (low vs high PD-1: mean 21.5 vs 33.3 months; p=0.010) and DMFS (low vs high PD-1: mean 19.3 vs 31.7 months; p=0.005) (Figure 2B and Table 3). Higher tumor grading adversely affected PFS (p=0.003), LPFS (p=0.022) and DMFS (p=0.002) but not OS (p=0.060). Univariate analyses also revealed a significant role was also observed for pT-stage, pN-stage, resection margins, perineural/neural invasion (PNI) and venous invasion (VI) with regard to all four clinical endpoints in the univariate analysis. We failed to detect a significant correlation for either total FOXP3 or PD-L1 expression with the clinical outcome.

**Table 3:** Univariate and multivariate analysis of prognostic factors

	Univariate	Multivariate			
	*p*-value	HR	95% CI		*p*-value
			Lower	Upper	
**OS**					
CD8 (Low *vs* High)	**0.037**	0.474	0.251	0.893	**0.021**
FOXP3 (Low *vs* High)	0.569	0.965	0.561	1.660	0.899
PD-1 (Low *vs* High)*	**0.028**	0.464	0.261	0.827	**0.049**
PD-L1 (Low *vs* High)	0.203	1.166	0.706	1.927	0.549
Age (<median(65) *vs* ≥median)	0.556	1.368	0.833	2.247	0.216
Sex (male *vs* female)	0.604	1.216	0.746	1.983	0.432
Tumour localisation (head *vs* other)		0.623	0.284	1.365	0.237
pT-stage (pT1-2 *vs* pT3-4)	**0.001**	0.550	0.323	0.935	**0.027**
pN-stage (pN0 *vs* pN+)	**0.001**	1.919	0.958	3.845	0.066
Grading (G1 *vs* G2 *vs* G3)	0.060	1.132	0.733	1.749	0.576
Resection margins (R0 *vs* R1)	**0.001**	1.268	0.710	2.264	0.423
Type of surgery (W *vs* PP *vs* TP)	0.848	1.153	0.750	1.772	0.517
PNI (no *vs* yes)	**0.001**	1.888	1.109	3.216	**0.019**
VI (no *vs* yes)	**0.006**	1.603	0.862	2.983	0.136
LI (no *vs* yes)	0.112	0.889	0.497	1.590	0.692
Chemotherapy (no *vs* 1-2 cycles *vs* ≥3 cycles)	**<0.001**	0.545	0.397	0.748	**0.001**
**PFS**					
CD8 (Low *vs* High)	**0.012**	0.556	0.313	0.988	**0.045**
FOXP3 (Low *vs* High)	0.839	0.902	0.534	1.524	0.700
PD-1 (Low *vs* High)*	**0.012**	0.652	0.398	1.068	0.089
PD-L1 (Low *vs* High)	0.436	1.038	0.642	1.679	0.878
Age (<median(65) *vs* ≥median)	0.617	1.293	0.822	2.035	0.267
Sex (male *vs* female)	0.753	1.145	0.715	1.833	0.574
Tumour localisation (head *vs* other)	0.311	0.758	0.374	1.540	0.444
pT-stage (pT1-2 *vs* pT3-4)	**0.001**	0.623	0.380	1.022	0.061
pN-stage (pN0 *vs* pN+)	**0.001**	2.455	1.288	4.679	**0.006**
Grading (G1 *vs* G2 *vs* G3)	**0.003**	1.533	1.021	2.303	0.040
Resection margins (R0 *vs* R1)	**0.001**	1.402	0.825	2.385	0.212
Type of surgery (W *vs* PP *vs* TP)	0.430	1.059	0.721	1.557	0.769
PNI (no *vs* yes)	**<0.001**	1.785	1.078	2.954	**0.024**
VI (no *vs* yes)	**0.005**	1.454	0.847	2.496	0.174
LI (no *vs* yes)	0.060	0.888	0.536	1.472	0.645
Chemotherapy (no *vs* 1-2 cycles *vs* ≥3 cycles)	**<0.001**	0.651	0.475	0.892	**0.008**
**LPFS**					
CD8 (Low *vs* High)	**0.046**	0.520	0.287	0.941	**0.031**
FOXP3 (Low *vs* High)	0.664	0.999	0.587	1.699	0.996
PD-1 (Low *vs* High)*	**0.010**	0.538	0.324	0.895	**0.017**
PD-L1 (Low *vs* High)	0.339	1.089	0.669	1.774	0.731
Age (<median(65) *vs* ≥median)	0.265	1.667	1.031	2.696	0.057
Sex (male *vs* female)	0.437	1.443	0.900	2.315	0.128
Tumour localisation (head *vs* other)	0.221	0.792	0.366	1.714	0.554
pT-stage (pT1-2 *vs* pT3-4)	**0.005**	0.725	0.433	1.213	0.221
pN-stage (pN0 *vs* pN+)	**0.001**	1.926	0.994	3.729	0.052
Grading (G1 *vs* G2 *vs* G3)	**0.022**	1.316	0.868	1.994	0.196
Resection margins (R0 *vs* R1)	**0.001**	1.564	0.910	2.690	0.106
Type of surgery (W *vs* PP *vs* TP)	0.545	1.192	0.780	1.820	0.417
PNI (no *vs* yes)	**<0.001**	2.028	1.214	3.388	**0.007**
VI (no *vs* yes)	**0.005**	1.508	0.857	2.655	0.155
LI (no *vs* yes)	0.083	0.853	0.499	1.456	0.559
Chemotherapy (no *vs* 1-2 cycles *vs* ≥3 cycles)	**<0.001**	0.529	0.385	0.728	**0.001**
**DMFS**					
CD8 (Low *vs* High)	**0.012**	0.393	0.207	0.746	**0.004**
FOXP3 (Low *vs* High)	0.756	0.917	0.534	1.575	0.754
PD-1 (Low *vs* High)*	**0.005**	0.540	0.320	0.910	**0.021**
PD-L1 (Low *vs* High)	0.479	1.015	0.615	1.676	0.953
Age (<median(65) *vs* ≥median)	0.914	1.223	0.762	1.963	0.404
Sex (male *vs* female)	0.438	1.256	0.769	2.050	0.362
Tumour localisation (head *vs* other)	0.316	0.643	0.306	1.349	0.242
pT-stage (pT1-2 *vs* pT3-4)	**0.001**	0.442	0.265	0.736	**0.002**
pN-stage (pN0 *vs* pN+)	**0.001**	2.414	1.240	4.699	**0.010**
Grading (G1 *vs* G2 *vs* G3)	**0.002**	1.350	0.883	2.065	0.166
Resection margins (R0 *vs* R1)	**0.001**	1.234	0.703	2.166	0.464
Type of surgery (W *vs* PP *vs* TP)	0.387	0.967	0.645	1.448	0.869
PNI (no *vs* yes)	**0.001**	1.834	1.088	3.090	**0.023**
VI (no *vs* yes)	**0.004**	1.625	0.918	2.879	0.096
LI (no *vs* yes)	0.076	0.895	0.528	1.518	0.681
Chemotherapy (no *vs* 1-2 cycles *vs* ≥3 cycles)	**<0.001**	0.540	0.460	0.895	**0.009**

Abbreviations: HR, hazard ratio; CI, confidence interval; W, Whipples; PP, partial pancreatectomy; TP, total pancreatectomy; VI, vascular invasion; LI, lymphatic invasion; PNI, perineural/neural invasion; OS, overall survival; PFS, progression-free survival; FFS, local failure-free survival; DMFS, distant metastases-free survival;

Significant values have been marked with bold.

Additionally, we conducted a multivariate analysis by including the immune markers and the clinicopathologic factors (Table 3). In the Cox model, high CD8 expression was confirmed as an independent prognostic parameter for OS (p=0.021), PFS (p=0.045), LPFS (p=0.031) and DMFS (p=0.004). In line to univariate analysis, higher PD-1+ TILs expression was associated with better OS (p=0.049), LPFS (p=0.017) and DMFS (p=0.021) but not PFS (p=0.089). For PD-1, a separate multivariate analysis was performed as, similarly to CD8, PD-1 also represents TILs. Adjuvant chemotherapy and PNI retained their significance for all four clinical endpoints in the multivariate analysis. Late pT-stage (pT3-4 vs pT1-2) correlated with worse OS (p=0.027) and DMFS (p=0.002), whereas lymph node metastases (pN+ vs pN0) were associated with worse PFS (p=0.006) and DMFS (p=0.010).

Moreover, we assessed the prognostic impact of the immune markers according to the three tumor compartments (intraepithelial, stroma and periphery; Table 4; Supplementary Figures 2-3). High stromal CD8+ and PD-1+ TILs expression predicted for better outcome, whereas PD-1+ TILs expression in the periphery and intraepithelial compartments associated with improved PFS and DMFS. Similarly to the total score, FOXP3 and PD-L1 expression in the different tumor compartments failed to predict for the clinical outcome in our analysis (Table 4).

**Table 4:** Prognostic impact of the immune markers in the different tumor compartments

TILs marker and tumour compartment	OS *p*-value	PFS *p*-value	LPFS *p*-value	DMFS *p*-value
CD8				
Tumor periphery	0.086	0.087	0.175	0.092
Tumor stroma	**0.021**	**0.034**	0.068	**0.004**
Tumor intraepithelial	0.632	0.320	0.142	0.567
FOXP3				
Tumor periphery	0.342	0.456	0.489	0.390
Tumor stroma	0.569	0.839	0.664	0.756
Tumor intraepithelial	0.721	0.541	0.289	0.367
PD-1				
Tumor periphery	0.253	**0.048**	0.121	**0.025**
Tumor stroma	**0.022**	**0.028**	**0.013**	**0.008**
Tumor intraepithelial	0.117	**0.027**	0.071	**0.020**
PD-L1				
Tumor periphery	0.162	0.305	0.286	0.479
Tumor stroma	0.259	0.307	0.259	0.475
Tumor intraepithelial	0.162	0.345	0.268	0.352

Abbreviations: OS, overall survival; progression-free survival; PFS, progression-free survival; LPFS, local progression-free survival; DMFS, distant metastases-free survival;

Significant values have been marked with bold.

Recently, tumors have been divided into four different groups based on the expression of TILs and PD-L1. Hence, we investigated the prognostic impact of these four groups (CD8^high^/PD-L1^high^ vs CD8^low^/PD-L1^low^ vs CD8^high^/PD-L1^low^ vs CD8^low^/PD-L1^high^) based on both the total and the stromal score of CD8 and PD-L1 (Supplementary Table 2; Supplementary Figure 4). Patients with CD8^high^/PD-L1^low^ expression in tumor stroma had a significantly superior OS (p=0.026), PFS (p=0.048) and DMFS (p=0.018), whereas a trend was found for better local control. The comparison of the four groups based on the total CD8 and PD-L1 score showed a trend towards significance for all four endpoints (Supplementary Table 2).

Furthermore, because the lack of prognostic value for FOXP3 was a surprising finding due to previous reports on their adverse impact in tumor progression, we performed an additional analysis whereby total and stromal CD8 and FOXP3 expression were combined together. In this analysis, we compared the clinical outcome of patients with high total and stromal CD8+/FOXP3- expression to the rest of the cohort (Supplementary Table 3; Supplementary Figure 5). Interestingly, the exclusion of tumors with FOXP3+ Tregs enhanced the prognostic significance of both total and stromal CD8+ TILs compared to the prognostic significance of CD8 alone.

### The correlation of immune markers with stromal morphology and lymphoid aggregates

We have recently reported that patients with high stromal density tumors have a significantly better outcome than patients with either moderate or loose density (20). Here, we examined the correlation of immune marker expression in the stromal compartment with stroma density (haematoxylin-eosin) and activated pancreatic stellate cells (alpha-smooth muscle actin, αSMA; Supplementary Table 4; Figure 1 and Supplementary Figure 1). Intriguingly, tumors of high stroma density had a significantly higher stromal expression of CD8+ TILs compared to that of moderate or loose density (p<0.001), whereas no correlation was found with αSMA. Additionally, some tumors with moderate or loose density stroma had higher CD8+ TILs expression in the tumor periphery/margin, indicating impaired penetration of these cells into the tumor as shown in Figure 1B. Of note, although tumors with high density had higher infiltration by CD8+ TILs compared to tumors with moderate or low density, nevertheless, infiltration was not homogeneous throughout the entire tumor surface area but rather heterogeneous. This finding emphasizes the importance of examining entire pancreatectomy sections as in our present work, rather than TMAs or small sections because the latter can lead to either under- or overestimation of histological measurements. None of the other immune markers showed significant association with the stroma morphology, indicating that not all TILs are PD-1 positive.

Finally, we found that 60 (41.4%) patients from the entire cohort (n=145) had tumor- associated intratumoral lymphoid aggregates that are similar to Crohn's-like lymphoid reaction pattern, ectopic lymph-node like structures, based on H&E staining. Hence, we examined the expression (absent vs present) of CD8+ TILs, PD-1+ TILs, FOXP3+ Tregs and PD-L1 in the lymphoid aggregates and its clinical impact (Supplementary Table 4; Supplementary Figure 6). The presence of PD-1+ TILs was associated with better OS (p=0.030), LPFS (p=0.025) and DMFS (p=0.033) but not PFS (p=0.075). CD8+ TILs only correlated with superior LPFS (p=0.039) and showed a trend towards better outcome for the other three clinical endpoints.

## DISCUSSION

Although previous studies have examined the impact of TILs and Tregs in PDAC on the clinical outcome of patients with PDAC, the prognostic value of PD-1 and PD-L1 and their association with TILs and Tregs remain largely unexplored in this disease. In the present work, patients with strong CD8+ TILs and PD-1+ TILs infiltration had a significantly better outcome compared to patients with low expression. This observation was independent of clinicopathologic parameters with a predictive role in this tumour type. In contrast to CD8 and PD-1, the prognostic impact of FOXP3+ Tregs and PD-L1+ cells was not significant.

CD8 is glycoprotein heterodimer of alpha and beta chains that are covalently linked by a disulfide bond and acts as a co-receptor for the T-cell receptor [13, 14]. CD8 binds to the major histocompatibility complex class I molecule together with the T-cell receptor to stimulate the cytotoxic effect of TILs on cancer cells and hence play an important role in cell-mediated immunity [13, 14]. In agreement with our data, several groups have shown superior survival in patients with high intratumoural CD8+ TILs expression in esophageal, colorectal, head and neck, breast, ovarian, renal, lung cancer and PDAC [13]. Despite the lack of clinical significance for FOXP3+ Tregs alone, their combination with CD8+ TILs (CD8+/FOXP3- vs other) further enhanced the prognostic value of CD8 in our series, which supports the notion that Tregs can contribute to tumor progression [15].

With regard to the prognostic impact of PD-1+ TILs, our observations are in line with previous studies showing a positive prognostic effect for PD-1+ TILs in patients with head and neck, ovarian cancer and colorectal cancer, spontaneously-regressing melanoma and folicullar lymphoma [16–19]. However, studies in other tumor types, including renal, nasopharyngeal and breast cancer have demonstrated an adverse role of PD-1+ TILs [20–22]. Several mechanisms have been proposed to explain this paradox. Antigen-specific immune activation following T-cell receptor stimulation leads to PD-1 upregulation on TILs [23]. Badoual et al. found higher expression of the immune activation markers HLA-DR and CD38 in PD-1+ TILs compared to PD-1- TILs [24]. Similarly, Ribas and colleagues revealed a strong positive predictive role for PD-1+ TILs following treatment with PD-1 inhibitors [25]. In contrast to melanoma non-responders, responders to immune checkpoint inhibitors had higher baseline tumor infiltration by CD8+ and PD-1+ T cells associated with PD-L1 upregulation in close tumor vicinity [25]. Additionally, PD-L1+ cells and PD-1+ TILs distribution were closely correlated, which is in line to preclinical studies showing PD-L1 to be upregulated upon release of IFNγ by PD-1+ TILs. The presence of PD-1+ TILs could thus reflect an endogenous antitumor immune response that occurred upon activation of TILs and although it led to decreased tumor growth, it failed to cause complete regression. Within this context, immune checkpoint inhibitors appear to be more effective in patients with pre-existing immunity suppressed by the PD-1/PD-L1 pathway that can be reinvigorated and reprogrammed with these agents [5].

We found tumor compartment-dependent differences in the prognostic value of CD8+ and PD-1+ TILs. Indeed, high stromal CD8+ and PD-1+ TILs infiltration was a positive prognostic factor for the clinical outcome. High PD-1+ but not CD8+ TILs infiltration in the intraepithelial and peripheral compartment correlated only with better PFS and DMFS. Mixed findings have been reported regarding the clinical significance of TILs infiltration according to the tumor compartment [26–29]. These differences could be attributed to the several factors, such as the heterogeneity in population and treatment administered but also the size of histological tissue samples and analysis method.

It is currently unclear whether PD-1 or PD-L1 can serve as reliable biomarkers for the prediction of response to immune checkpoint inhibitors. Recently, four different tumor groups were proposed according to the combined TILs and PD-L1 status (positive or negative) in melanoma to facilitate future immunotherapy decisions [30]. These include type I (TILs+/PD-L1+ mediating adaptive immune resistance), type II (TILs-/PD-L1- mediating immunologic ignorance), type III (TILs-/PD-L1- mediating intrinsic induction), and type IV (TILs+/PD-L1- mediating tolerance). In the present study, patients with type IV PDAC (CD8^high^/PD-L1^low^), based on the stromal expression, had superior outcome compared to the other groups. Thompson et al. also classified their cohort into the four groups and observed higher CD8+ TILs infiltration in tumors PD-L1+ compared to PD-L1- gastric cancer indicating an active adaptive immune resistance mechanism [31]. However, the clinical impact of the four groups was not reported in their work. More reports in other tumor types are needed before routine use of this four-group classification.

Although work have assessed the prognostic impact of TILs and Tregs, and recently of PD-L1 [32], in PDAC, nevertheless, none of these studies examined in detail the association of all four immune markers with the desmoplastic stoma and their localization in large pancreatectomy sections. Traditionally, the abundant desmoplastic stroma has been regarded as a barrier impairing tumor infiltration by TILs [3, 4]. Ino et al. previously reported better outcome in patients with high stromal expression of CD8 [11], which is in agreement with our data but a recent work failed to detect a prognostic role for CD8+ TILs [32]. Also, even in tumors with high CD8+ TILs infiltration in the stroma, the majority of tumors lacked CD8+ TILs in the close proximity to cancer cells in our cohort. Fearon and colleagues have previously demonstrated increased TILs infiltration and improved response to anti-PD-L1 following blockade of CXCR4/CXCL12 signaling mediated by FAP+ stromal cells in the KPC mouse model [12]. Additional mechanisms have been implicated in the exclusion of TILs from PDAC. These include the presence of immunosuppressive cells, such as neutrophils and macrophages that reside both inside and outside the tumor [33, 34], activated pancreatic stellate cells that drive apoptosis and sequestration of TILs [10], and the physical barrier imposed by the stroma. Relevant to the latter, preclinical studies have reported higher TILs motility and migration in tumor areas with loose collagen and vice versa [35]. Analysis of CD8+ TILs expression showed significantly higher infiltration in patients with strong stroma density compared to patients with loose stroma density and vice versa in our cohort. In accordance, Ueno et al. also found higher CD8+ TILs expression in colorectal tumors with high stromal density that was decreased according to the decrease in stroma density [36]. These results suggest that the stroma density affects TILs guidance. We noted lack of homogeneous distribution of CD8+ TILs throughout the entire tumor surface area even in the high stroma density tumors that presented significantly higher TILs infiltration compared to tumors with moderate or low stroma density. It remains unclear whether formation of the highly-dense stroma either precedes, parallels or follows TILs infiltration.

Lymphoid aggregates are lymphoid-like structures that often develop at inflammatory sites including cancer, infection and autoimmune diseases [37]. Their structure varies from clusters of T and B cells to germinal-like centers. Their clinical relevance remains controversial as both positive and negative prognostic outcomes have been reported in patients with lymphoid aggregates [37]. The prognostic role of these structures remains largely unknown in PDAC. In contrast to our data, Lutz et al. reported lack of lymphoid aggregates in 54 primarily resected PDAC specimens obtained from previously untreated patients [38]. Interestingly, they only observed aggregates in 33 out of the 39 patients 2 weeks after administration of a GVAX vaccine that was associated with prolonged survival in some but not all patients. Lymphoid aggregates expressed both immunosuppressive and proinflammatory signals. Also, PD-1 and PD-L1 were expressed in all aggregates, whereas lower expression of PD-L1 within the aggregates was linked to superior OS [38]. In line to the total expression data, only patients with higher expression of CD8+ and PD-1+ TILs in the lymphoid aggregates demonstrated longer OS in our series. However, further reports are missing in the literature, possibly due to the fact that the vast majority of the pathology studies to date have been conducted in TMAs. Further investigations in large tumor sections are needed to elucidate the biological role of aggregates in PDAC.

We would like to acknowledge the limitations of the study. First, although patients were treated and followed up prospectively, the retrospective analysis could have resulted in selection bias. Second, the median follow-up in our study is relatively short as studies with longer follow-up have been previously reported. Finally, our findings warrant validation in prospective cohorts using detailed pathology protocol.

To summarize, CD8+ and PD-1+ TILs represent strong prognostic marker to identify PDAC patients with a more favorable outcome. Interestingly, TILs infiltration was more pronounced in patients with highly-dense mature stroma compared to patients with lower density stroma, whereas TILs localization was heterogeneous. The expression of these markers in lymphoid aggregates also correlated with a superior survival. CD8 and PD-1 can be used as surrogate markers to predict clinical outcome and could be potentially exploited in future studies to guide novel immunotherapies that stimulate T-cell-activity.

## MATERIALS AND METHODS

### Patients and treatment

Patients with PDAC received surgery followed by postoperative chemotherapy in the period between 2009 and 2014 at the Oxford University Hospital NHS Trust, Oxford, UK. The type of pancreatectomy performed was based on international guidelines. Patients that met the following criteria were included in this retrospective analysis: histologically-confirmed PDAC, previous complete macroscopic surgical resection (R0 or R1), absence of distant metastases and/or ascites, absence of other malignancies, no previous treatment, and availability of formalin-fixed paraffin-embedded (FFPE) tissue samples in the pathology archive. In total, n=145 patients that fulfilled all criteria were included in the present study. The majority of patients received gemcitabine (GEM) as monotherapy. GEM alone was given intravenously at a dose of 1,000 mg/m^2^ over 30 minutes, at days 1, 8 and 15 (1 cycle) for a total of 6 cycles. Few patients were treated with a combination of gemcitabine with capecitabine (GEM-CAP). In this case, GEM was administered as described above, whereas CAP was given orally at a dose 1,660 mg/m^2^/d (830 mg/m^2^ twice daily) for 3 weeks followed by 1 week pause. FFPE tissue blocks were obtained from the Pathology archive together with clinical follow-up data and diagnostic images at the Oxford University Hospital NHS Trust. Informed consent had been previously obtained. The present study was approved by the Oxford Radcliffe Biobank, University of Oxford (local ethics committee Project: OCHRe 14/A176).

### Immunohistochemical staining and scoring

All slides from the n=145 pancreatomy samples were reviewed by an experienced pathologist and the best representative FFPE tissue block (highest cellularity, most representative of stromal morphology, least amount of necrosis) was selected, and 3-μm thick sections were cut and mounted on coated superfrost slides. Slides were stained with haematoxylin and eosin (H&E) as described before [39]. For the immunohistochemical staining we used the Leica Bond Max staining platform, Department of Pathology, Oxford University Hospital NHS Trust. A horseradish-peroxidase technique and a DAKO Autostainer Link 48 (DAKO, UK) were used. Antibody detection was achieved using the Leica DS 9800 detection system. Antigen retrieval was performed automatically by the pretreatment of the paraffin sections (SuperFrost Plus, Thermo Scientific, UK) using either Bond ER 1 (Citrate based buffer at Ph 6) or Bond ER2 (EDTA based buffer at Ph 9; both Leica Microsystems, UK) for 20 min on the Bond Max staining machine. Subsequently, the slides were stained with the primary antibodies for either CD8 (1:100, clone C8/144B; Dako M7103, UK), FOXP3 (1:100, clone 236A/E7; Abcam AB20034, UK), PD-1 (1:80, clone NAT105; Cell Marque CMC31529030, UK) and PD-L1 (1:200, clone E1L3N(R); Cell Signal technology, UK) following incubation for 180 minutes at room temperature. Following this, dextran polymer-conjugated horseradish-peroxidase and 3,3′-diaminobenzidine (DAB) chromogen intensified with 1 % copper sulphate was applied followed by a light haematoxylin counterstain (Gill 3, Sigma, UK).

Scoring for the expression of CD8+ TILs and PD-1+ TILs as well as FOXP3+ Tregs was performed semiquantitatively by measuring cell density as previously reported [26, 40]. Scoring was as follows: (1) absent cells; (2) <25% cell density; (3) 25-50% cell density; (4) >50% cell density. Cells were assessed in all three compartments of the tumour: the intra-epithelial compartment (cells within and in direct contact with tumour cell nests); the stroma (cells within the intratumoural stroma) and the tumour periphery (cells localised in tumour periphery). The sum of the separate scores from the three tumour compartments (intra-epithelial compartment, stroma and tumour periphery) determined the total score for CD8+, PD-1+ and FOXP3+ cells. The total score ranged from 3 to 12. We used the median score value as a cut-off to classify patients into two groups: low or high CD8+, PD-1+ and FOXP3+ cells expression. Similarly to other markers, PD-L1+ tumor or myeloid cells was also examined in all 3 tumour compartments and scored as shown above. Furthermore, we investigated the prognostic value of the markers for each of the 3 different compartments separately. Additionally, we evaluated the prognostic impact of the TIL score for each of the three different tumour compartments (intraepithelial, stroma and periphery). For that purpose, the median score of each area was measured and the cut-off point was chosen to separate the cohort into two subgroups with either low or high score. Stromal density was classified as loose, moderate or strong, whereas αSMA was scored as high, moderate and low as recently reported [41]. With regard to lymphoid aggregates, either the presence or absence of CD8, FOXP3, PD-1 and PD-L1 expression was considered for estimating their prognostic value.

The ImageScope Viewer (Aperio Technologies, Inc., Vista, CA, USA) was used to scan and analyse slides. At least ten different areas of the tumor were assessed. Immunohistochemical scoring was established by a board certified pathologist (LMW) with expertise in gastrointestinal malignancies. Blinded samples were evaluated by two observers (LMW and EF). A final decision and consensus was made after additional examination of the specimens in cases of discrepancy. The dominant staining pattern was used for scoring in cases of extensive intratumoral heterogeneity. Representative images from patients with different expression of the four immune markers together with H&E (stroma density) and αSMA (stroma activity) are shown in Figure 1 and Suppelementary Figure 1.

### Statistics

We used the Fisher's exact test to examine the differences between categorical variables. Overall survival (OS) was measured from the date of surgery to the day of death from any cause. Progression-free survival (PFS) was calculated from the date of surgery to the day of local or distant recurrence or death from any cause. Distant metastasis free survival (DMFS) and local progression free survival (LPFS) were measured from the date of surgery to distant metastasis or death, and local progress or death, respectively. Patients that did not develop either local or distant tumor recurrence were censored at the last follow-up contact. A p-value lower than 0.05 was considered statistically significant. Survival curves were plotted with the Kaplan–Meier method. Univariate analyses were conducted with the log-rank (Mantel–Cox) test and multivariate analyses with the Cox proportional hazard model. All statistical analyses were performed using the SPSS 20 software (SPSS Inc., Chicago, IL, USA).

## SUPPLEMENTARY MATERIALS FIGURES AND TABLES


