# Supplementary material for: Prognostic value, localization and correlation of PD-1/PD-L1, CD8 and FOXP3 with the desmoplastic stroma in pancreatic ductal adenocarcinoma
Source: Oncotarget. 2016 Jun 14;7(27):40992–1004. doi: 10.18632/oncotarget.10038 (PMC5173037; doi:10.18632/oncotarget.10038)
Supplement: Supplementary file 1 [file oncotarget-07-40992-s001.pdf]

Prognostic value, localization and correlation of PD-1/PD-L1, CD8 and FOXP3 with the desmoplastic stroma in pancreatic ductal adenocarcinoma

Supplementary Material

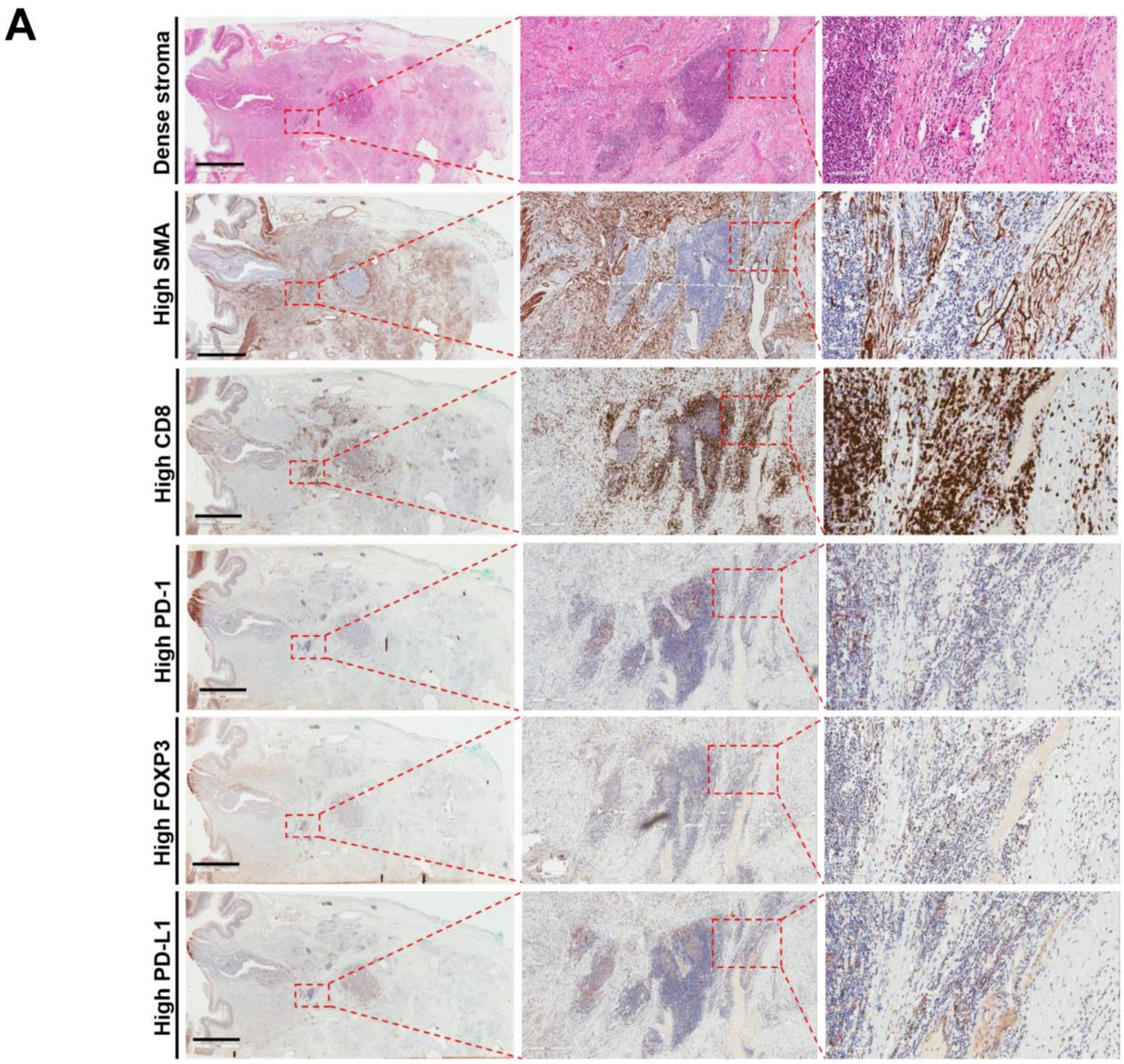

B

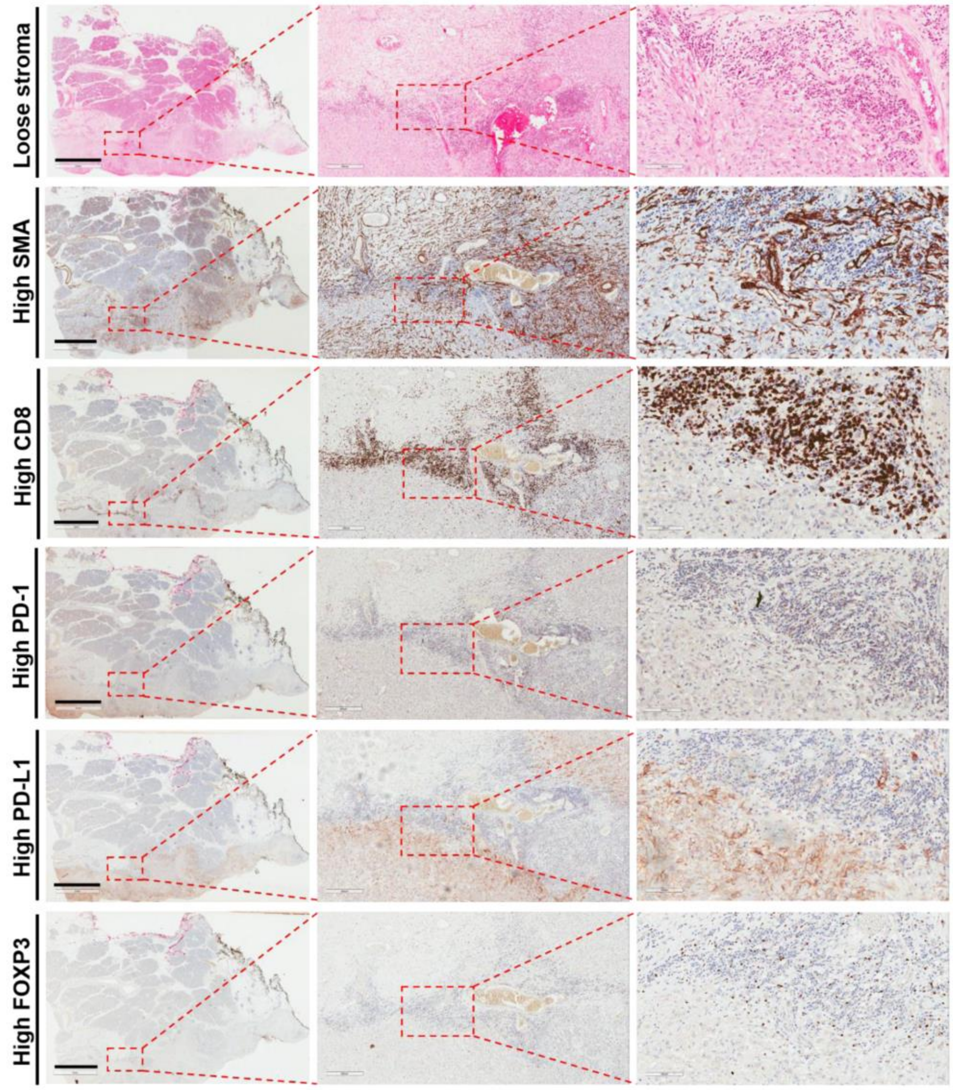

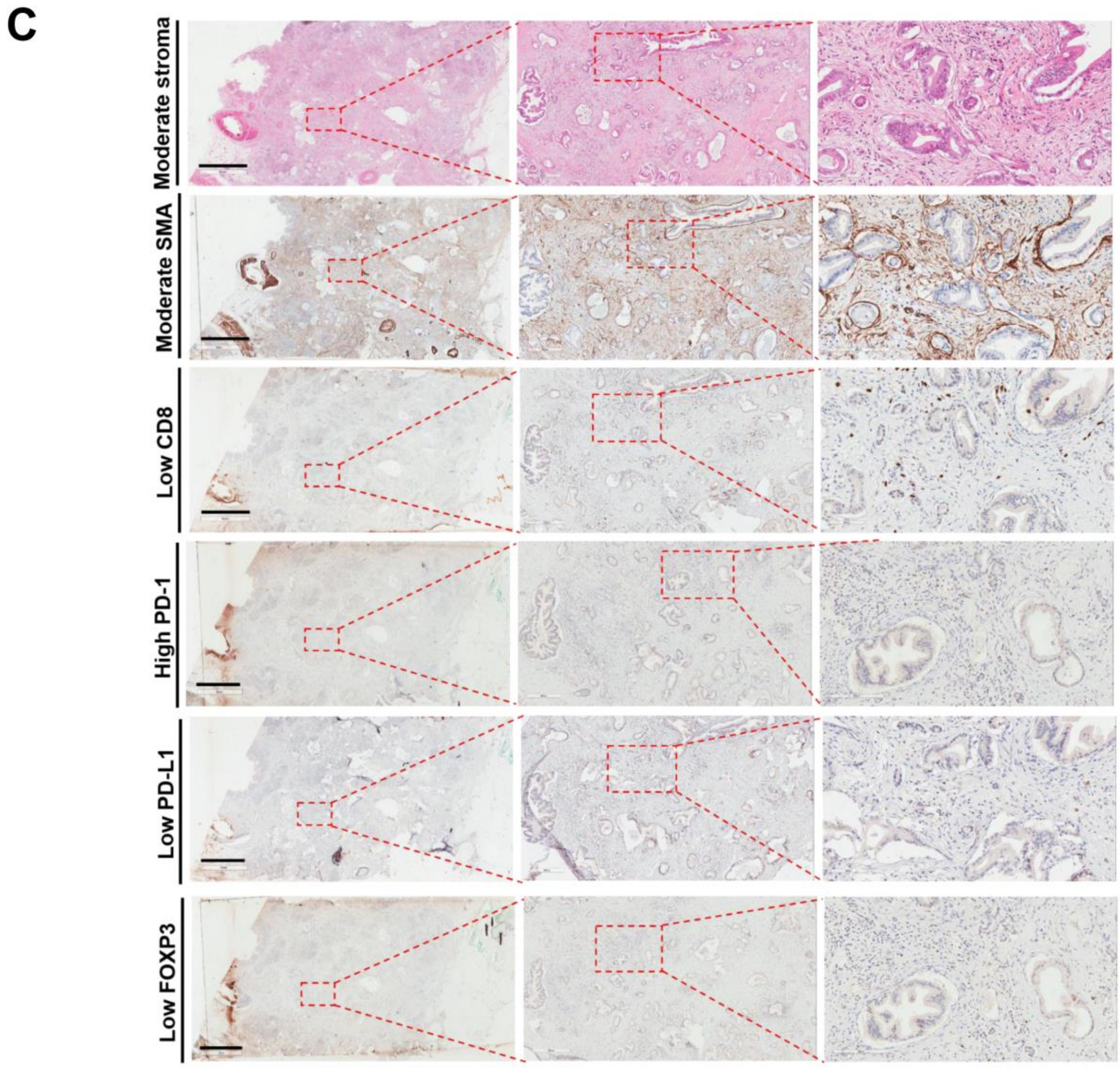

Supplementary Figure 1. Representative examples (same sections as in main Figure 1) of CD8+ TILs, PD-1+ TILs, PD-L1 tumor (and myeloid cells) and FOXP3+ Tregs in patients with pancreatic cancer adenocarcinoma with ***(A)*** high-density, ***(B)*** moderate density, and ***(C)*** low density stroma based on H&E staining, as indicated. The corresponding αSMA images are shown as well. The left panels illustrate large pancreatectomy sections (Bar: 5 mm). The magnifications of the middle and right inserts are x50 and x200, respectively.

A

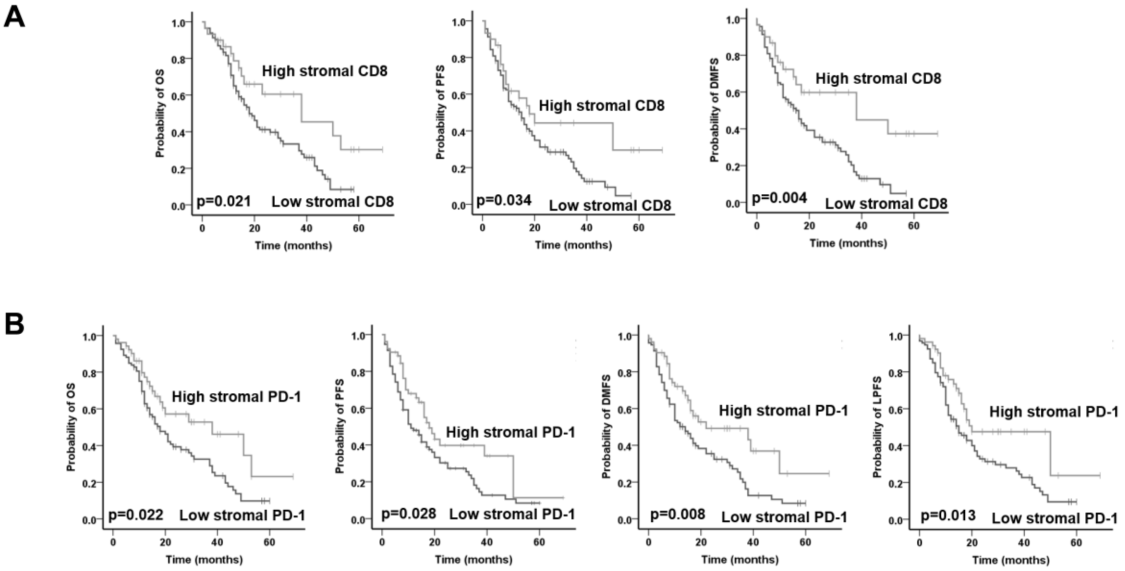

Supplementary Figure 2. Prognostic impact of stromal (A) CD8+ TILs and (B) PD-1+ TILs on overall survival (OS), progression-free survival (PFS), local progression-free survival (LPFS) and distant metastases free survival (DMFS) in patients with pancreatic cancer adenocarcinoma, as indicated. Only plots from significant results are shown here. Analysis was based on the dichotomized stromal CD8 and PD-1 score in resected patient samples (cut-off according to median value of the corresponding score).

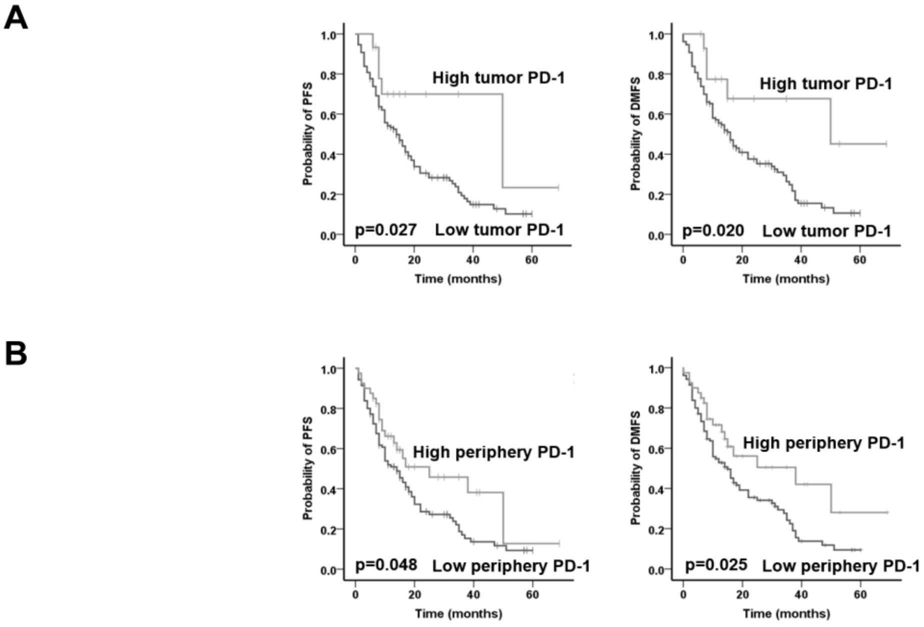

Supplementary Figure 3. Prognostic impact of PD-1+ TILs in the (A) tumor (intraepithelial) compartment and (B) tumor periphery on progression-free survival (PFS) and distant metastases free survival (DMFS) in patients with pancreatic cancer adenocarcinoma, as indicated. Only plots from significant results are shown here. Analysis was based on the dichotomized intraepithelial and tumor periphery PD-1 score in resected patient samples (cut-off according to median value of the corresponding score).

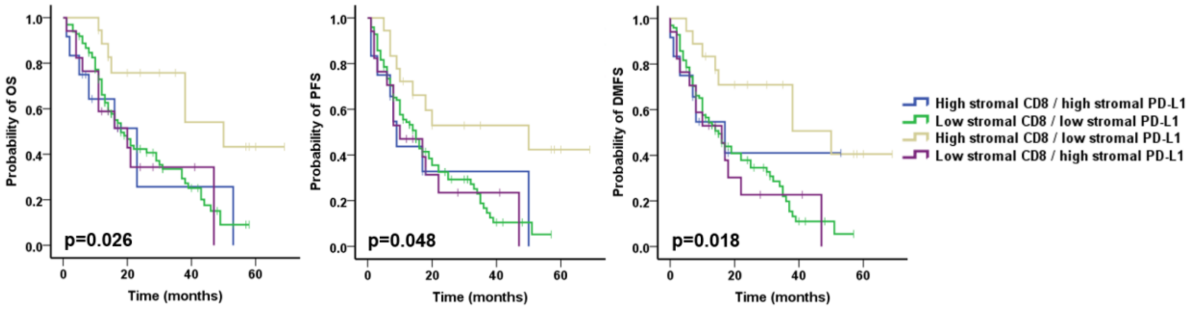

Supplementary Figure 4. Prognostic impact of the four groups ($CD8^{high}/PD\text{-}L1^{high}$ vs $CD8^{low}/PD\text{-}L1^{low}$ vs $CD8^{high}/PD\text{-}L1^{low}$ vs $CD8^{low}/PD\text{-}L1^{high}$) based on stromal score of CD8 and PD-L1 on overall survival (OS), progression-free survival (PFS) and distant metastases free survival (DMFS) in patients with pancreatic cancer adenocarcinoma, as indicated. Analysis was based on the dichotomized stromal CD8 and PD-L1 score in resected patient samples (cut-off according to median value of the corresponding score).

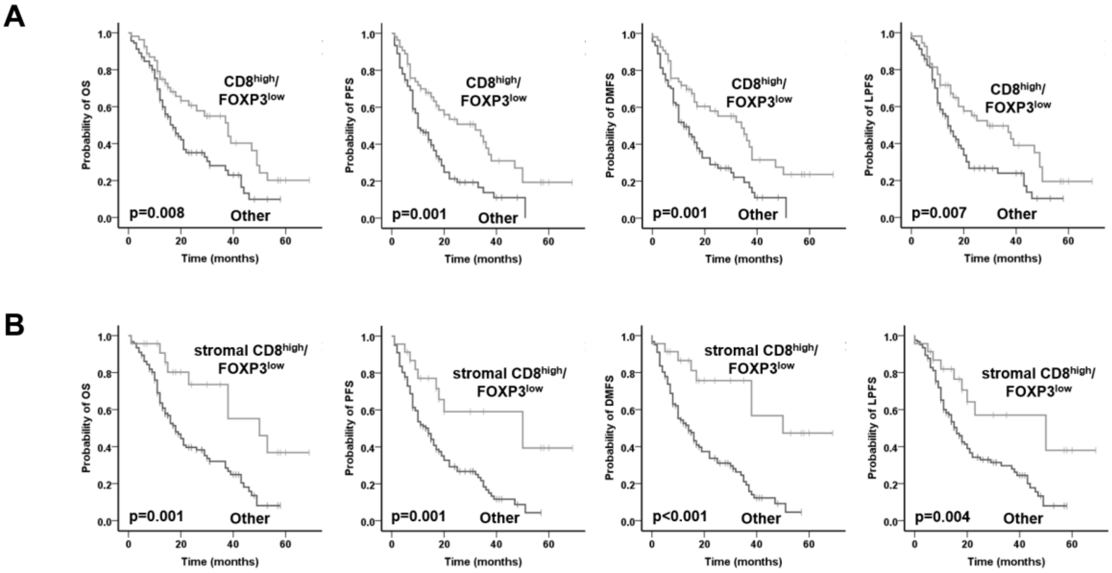

Supplementary Figure 5. Prognostic impact of ***(A)*** total and ***(B)*** stromal $CD8^{high}$ / $FOXP3^{low}$ vs Other on overall survival (OS), progression-free survival (PFS), local progression-free survival (LPFS) and distant metastases free survival (DMFS) in patients with pancreatic cancer adenocarcinoma, as indicated. Analysis was based on the dichotomized total and stromal CD8 and FOXP3 score in resected patient samples (cut-off according to median value of the corresponding score).

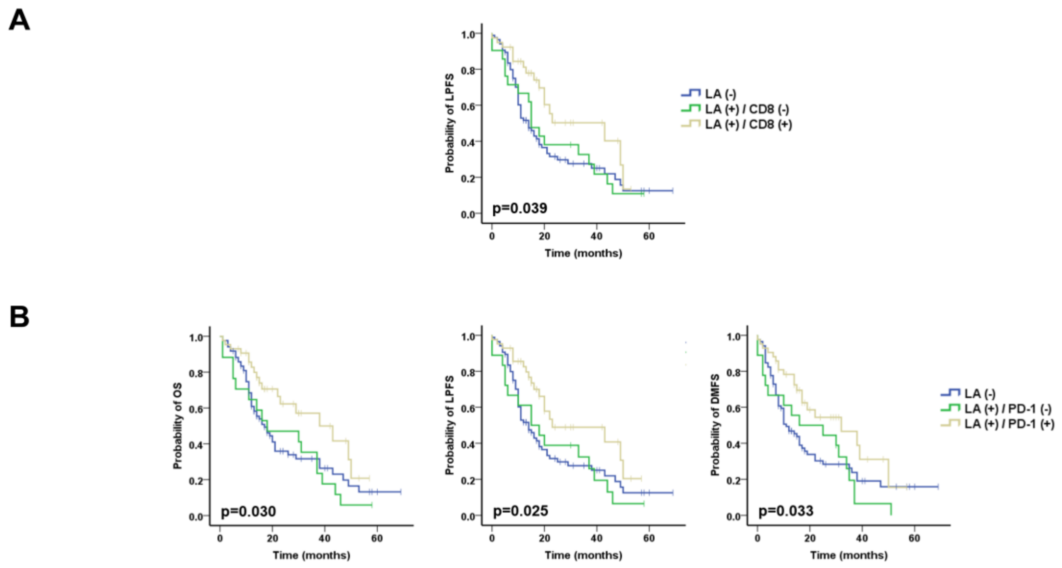

Supplementary Figure 6. Prognostic impact of the presence of ***(A)*** CD8+ TILs and ***(B)*** PD-1+ TILs in lymphoid aggregates (LA) for overall survival (OS), local progression-free survival (LPFS) and distant metastases free survival (DMFS) in patients with pancreatic cancer adenocarcinoma, as indicated. Only plots from significant results are shown here. Analysis was based on the either the presence (+) or absence (-) of CD8+ and PD-1+ TILs in LA.

Supplementary Table 1. Clinicopathological characteristics of the cohort

	n (%)
**Age**	
<median (65 years)	63 (43.4%)
≥median	82 (56.6%)
**Gender**	
Male	68 (46.9%)
Female	77 (53.1%)
**Tumor site**	
Head	120 (82.8%)
Other	25 (17.2%)
**pT-staging**	
pT1-2	88 (60.7%)
pT3-4	57 (39.3%)
**pN-staging**	
pN0	35 (24.1%)
pN+	110 (75.9%)
**Grading**	
G1	8 (5.5%)
G2	94 (64.8%)
G3	43 (29.7%)
**Resection margins**	
R0	54 (37.2%)
R1	91 (62.8%)
**Type of surgery**	
Whipples	92 (63.4%)
Pylorus preserving	38 (26.2%)
Total pancreatectomy	15 (10.4%)
**PNI**	
no	114 (78.6%)
yes	31 (21.4%)
**VI**	
no	52 (35.9%)
yes	93 (64.1%)
**LI**	
no	53 (36.6%)
yes	92 (63.4%)
**Chemotherapy**	
No	19 (13.1%)
1-2 cycles	32 (22.1%)
≥3 cycles	94 (64.8%)

Abbreviations: VI, vascular invasion; LI, lymphatic invasion; PNI, perineural/neural invasion;

Supplementary Table 2. Prognostic influence of total and stromal CD8 combined with PD-L1 on the clinical outcome of patients

Marker	OS p-value	PFS p-value	LPFS p-value	DMFS p-value
Total $CD8^{high}$ / PD-$L1^{high}$ vs $CD8^{low}$ / PD-$L1^{low}$ vs $CD8^{high}$ / PD-$L1^{low}$ vs $CD8^{low}$ / PD-$L1^{high}$	0.071	0.065	0.111	0.080
Stromal $CD8^{high}$ / PD-$L1^{high}$ vs $CD8^{low}$ / PD-$L1^{low}$ vs $CD8^{high}$ / PD-$L1^{low}$ vs $CD8^{low}$ / PD-$L1^{high}$	**0.026**	**0.048**	0.068	**0.018**

Abbreviations: OS, overall survival; PFS, progression-free survival; LPFS, local progression-free survival; DMFS, distant metastases-free survival;

*Significant values have been marked in bold.

Supplementary Table 3. Prognostic influence of total and stromal CD8 in conjunction with FOXP3 on the clinical outcome of patients

Marker	OS p-value	PFS p-value	LPFS p-value	DMFS p-value
Total $CD8^{high}$ / $FOXP3^{low}$ vs Other	**0.008**	**0.001**	**0.001**	**0.007**
Stromal $CD8^{high}$ / $FOXP3^{low}$ vs Other	**0.001**	**0.001**	**<0.001**	**0.004**

Abbreviations: TILs, tumor infiltrating lymphocytes; OS, overall survival; PFS, progression-free survival; LPFS, local progression-free survival; DMFS, distant metastases-free survival;

Significant values have been marked in bold.

Supplementary Table 4. The correlation of immune marker infiltration in the stromal compartment with stroma density and αSMA

	Low CD8 n (%)	High CD8 n (%)	p-value	Low FOXP3 n (%)	High FOXP3 n (%)	p-value	Low PD-1 n (%)	High PD-1 n (%)	p-value	Low PD-L1 n (%)	High PD-L1 n (%)	p-value
**Stroma density**												
Loose	29 (25.2%)	1 (3.3%)	**<0.001**	11 (28.9%)	19 (17.8%)	0.310	21 (22.6%)	9 (17.3%)	0.557	28 (24.1%)	2 (6.9%)	0.108
Moderate	73 (63.5%)	17 (56.7%)		22 (57.9%)	68 (63.6%)		58 (62.4%)	32 (61.5%)		68 (58.6%)	22 (75.9%)	
Strong	13 (11.3%)	12 (40%)		5 (13.2%)	20 (18.7%)		14 (15.1%)	11 (21.2%)		20 (17.2%)	5 (17.2%)	
**αSMA**												
Negative+Weak	25 (21.7%)	7 (23.3%)	0.851	7 (18.4%)	25 (23.4%)	0.528	19 (20.4%)	13 (25%)	0.525	27 (23.3%)	5 (17.2%)	0.483
Moderate+Strong	90 (78.3%)	23 (76.7%)		31 (81.6%)	82 (76.6%)		74 (79.6%)	30 (75%)		89 (76.7%)	24 (82.8%)	

Supplementary Table 5. Expression of CD8, FOXP3, PD-1 and PD-L1 in the n=60 patients with lymphoid aggregates and its impact on the clinical outcome

**Immune marker**	**CD8 N (%)**	**FOXP3 N (%)**	**PD-1 N(%)**	**PD-L1 N (%)**
Absent	21 (35%)	42 (70%)	18 (30%)	15 (25%)
Present	39 (65%)	18 (30%)	42 (70%)	45 (75%)
OS p-value	0.075	0.261	**0.030**	0.274
PFS p-value	0.145	0.319	0.075	0.334
LPFS p-value	**0.039**	0.155	**0.025**	0.172
DMFS p-value	0.068	0.270	**0.033**	0.274

Abbreviations: OS, overall survival; PFS, progression-free survival; FFS, local failure-free survival; DMFS, distant metastases-free survival;

Significant values have been marked with bold.
